# cAMP‐Induced Nuclear Condensation of CRTC2 Promotes Transcription Elongation and Cystogenesis in Autosomal Dominant Polycystic Kidney Disease

**DOI:** 10.1002/advs.202104578

**Published:** 2022-01-17

**Authors:** Zeyun Mi, Yandong Song, Jiuchen Wang, Zhiheng Liu, Xinyi Cao, Lin Dang, Yumei Lu, Yongzhan Sun, Hui Xiong, Lirong Zhang, Yupeng Chen

**Affiliations:** ^1^ Key Laboratory of Immune Microenvironment and Disease (Ministry of Education) The Province and Ministry Co‐sponsored Collaborative Innovation Center for Medical Epigenetics Department of Biochemistry and Molecular Biology School of Basic Medical Sciences Tianjin Institute of Urology The Second Hospital of Tianjin Medical University Tianjin Medical University Tianjin 300070 China; ^2^ Department of Urology Shandong Provincial Hospital Affiliated to Shandong First Medical University Jinan Shandong 250001 China

**Keywords:** ADPKD, cAMP, condensate, CRTC2, phase separation, P‐TEFb

## Abstract

Formation of biomolecular condensates by phase separation has recently emerged as a new principle for regulating gene expression in response to extracellular signaling. However, the molecular mechanisms underlying the coupling of signal transduction and gene activation through condensate formation, and how dysregulation of these mechanisms contributes to disease progression, remain elusive. Here, the authors report that CREB‐regulated transcription coactivator 2 (CRTC2) translocates to the nucleus and forms phase‐separated condensates upon activation of cAMP signaling. They show that intranuclear CRTC2 interacts with positive transcription elongation factor b (P‐TEFb) and activates P‐TEFb by disrupting the inhibitory 7SK snRNP complex. Aberrantly elevated cAMP signaling plays central roles in the development of autosomal dominant polycystic kidney disease (ADPKD). They find that CRTC2 localizes to the nucleus and forms condensates in cystic epithelial cells of both mouse and human ADPKD kidneys. Genetic depletion of CRTC2 suppresses cyst growth in an orthologous ADPKD mouse model. Using integrative transcriptomic and cistromic analyses, they identify CRTC2‐regulated cystogenesis‐associated genes, whose activation depends on CRTC2 condensate‐facilitated P‐TEFb recruitment and the release of paused RNA polymerase II. Together, their findings elucidate a mechanism by which CRTC2 nuclear condensation conveys cAMP signaling to transcription elongation activation and thereby promotes cystogenesis in ADPKD.

## Graphical abstract

Biomolecular condensates play key roles in controlling gene expression in response to internal and external signals. This study elucidates a mechanism by which CRTC2 nuclear condensation conveys cAMP signaling to transcription elongation activation and cystogenesis in autosomal dominant polycystic kidney disease.

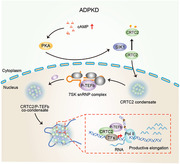

## 1 Introduction

Biomolecular condensates are membraneless bodies that often form through liquid–liquid phase separation by compartmentalizing functionally related protein and DNA/RNA molecules.^[^ ^1^, ^2^ ^]^ Accumulating evidence indicates that phase‐separated condensates are involved in many cellular processes, including cellular signal transduction and regulation of gene expression.^[^ ^3^, ^4^, ^5^, ^6^ ^]^ In addition, aberrant forms of phase‐separated condensates have been reported in diverse human diseases, such as neurodegeneration and cancers.^[^ ^7^, ^8^, ^9^, ^10^ ^]^ Alterations in signaling pathways in disease lead to changes in gene expression programs, but whether and how phase separation is involved in the regulation of cellular gene expression by disease‐perturbed signaling pathways remain largely unknown.

Adenosine 3',5'‐cyclic monophosphate (cAMP) is a critical intracellular second messenger that mediates the intracellular response to neurotransmitters and multiple hormones.^[^ ^11^, ^12^ ^]^ cAMP signaling can trigger a plethora of cellular effects, including gene expression,^[^ ^13^, ^14^ ^]^ cell proliferation and differentiation,^[^ ^15^, ^16^ ^]^ apoptosis,^[^ ^17^, ^18^ ^]^ and diverse metabolic processes, such as insulin and glucagon secretion,^[^ ^19^ ^]^ gluconeogenesis,^[^ ^20^, ^21^ ^]^ adipogenesis, and lipolysis.^[^ ^22^ ^]^ Alterations in cAMP signaling affect gene expression by modulating the activity of several cAMP‐responsive transcription effectors, including cAMP response element‐binding protein (CREB),^[^ ^23^, ^24^, ^25^ ^]^ cAMP‐responsive element modulator (CREM),^[^ ^26^ ^]^ activating transcription factor 1 (ATF1),^[^ ^27^ ^]^ and CREB‐regulated transcription coactivators (CRTCs).^[^ ^28^, ^29^, ^30^, ^31^, ^32^ ^]^ Recently, we reported that cAMP signaling can also activate a general transcription regulator, the positive transcription elongation factor b (P‐TEFb).^[^ ^33^ ^]^ P‐TEFb is a protein complex consisting of a kinase subunit, CDK9, and a regulatory subunit, CycT1.^[^ ^34^ ^]^ P‐TEFb activity is essential for gene activation, particularly for developmental and signal‐responsive genes, whose promoter‐proximal regions are frequently pre‐loaded with paused RNA polymerase II (Pol II) before signal stimulation.^[^ ^35^, ^36^ ^]^ Activated P‐TEFb promotes transcription elongation of these genes by phosphorylating the C‐terminal domain (CTD) of the largest subunit of Pol II and negative elongation factors, triggering the release of paused Pol II to productive elongation.^[^ ^37^, ^38^ ^]^

Aberrant activation of the cAMP pathway plays central roles in the pathogenesis of autosomal dominant polycystic kidney disease (ADPKD), an inherited kidney disease characterized by numerous fluid‐filled renal cysts and impaired renal function.^[^ ^39^, ^40^, ^41^, ^42^, ^43^, ^44^ ^]^ We previously found that P‐TEFb was aberrantly activated in ADPKD cells, which resulted in increased expression of genes related to cystogenesis.^[^ ^33^ ^]^ P‐TEFb is thought to be critical for the release of paused Pol II at almost all metazoan genes.^[^ ^35^, ^38^, ^45^ ^]^ However, aberrant activation of P‐TEFb in ADPKD cells caused increased expression in only a subset of genes.^[^ ^33^ ^]^ The mechanisms by which P‐TEFb is specifically recruited to the cystogenesis‐related genes and activates this gene‐specific transcription elongation in response to cAMP signaling are unclear.

Here we report that cAMP induces CRTC2 nuclear translocation and condensate formation, which leads to interaction and co‐condensation with the P‐TEFb complex. CRTC2 nuclear condensates extract P‐TEFb from the inhibitory 7SK snRNP complex. Aberrant CRTC2 condensates form in cystic renal epithelial cells, and are required for the recruitment of P‐TEFb to activate transcription elongation of genes related to cystogenesis. Thus, we propose that phase separation plays a key role in coordinating cAMP signaling for gene regulation by generating condensates that concentrate both on signal‐specific effectors and general transcription regulators. Dysregulation of this mechanism may underlie the pathological gene expression in ADPKD.

## 2 Results

### 2.1 cAMP Signaling Induces CRTC2 Nuclear Condensation

**Figure 1:**
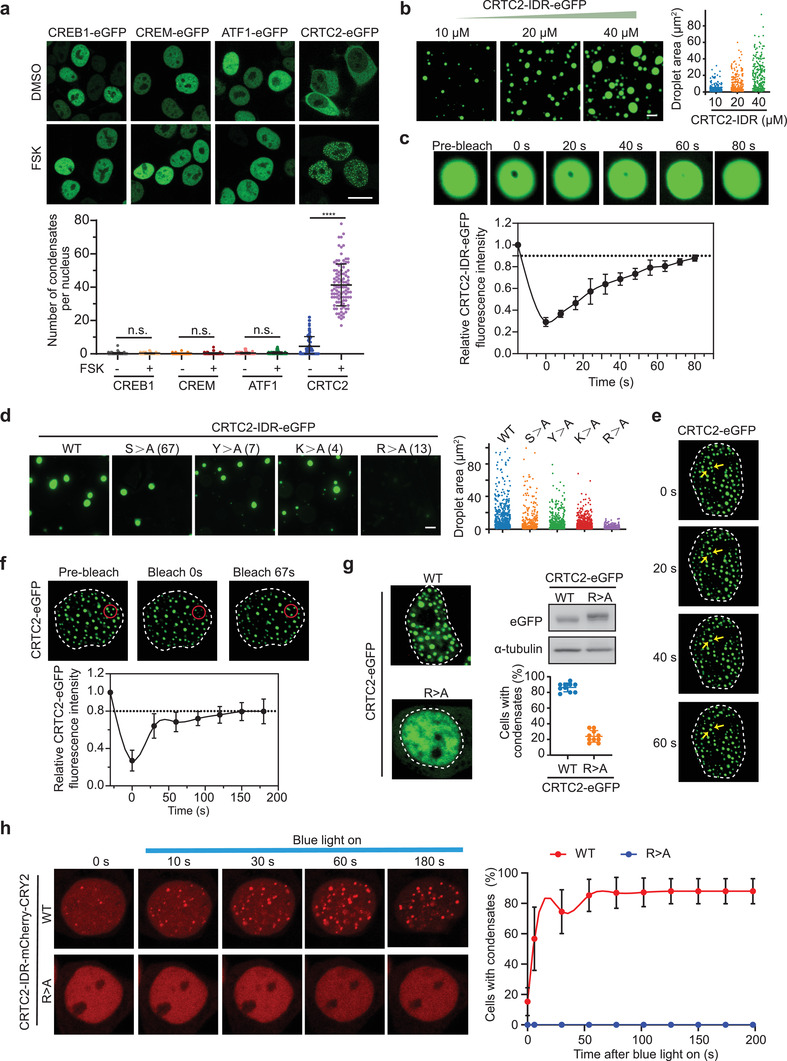
cAMP signaling induces CRTC2 nuclear translocation and condensate formation. a) Live‐cell imaging of ectopically expressed CRTC2‐eGFP, CREB‐eGFP, CREM‐eGFP, and ATF1‐eGFP in 293T cells without (DMSO) and with forskolin (FSK) treatment (upper). Quantification of condensates number per nucleus (lower). b) In vitro droplet formation assay with recombinant CRTC2‐IDR‐eGFP at different protein concentrations (left). Quantification of the size of droplets (right). c) Representative images of the in vitro FRAP experiment with recombinant CRTC2‐IDR‐eGFP (upper). Quantification of FRAP data for CRTC2‐IDR‐eGFP puncta (lower). d) In vitro droplet formation assay of recombinant eGFP fusion proteins fused with wild‐type (WT) CRTC2‐IDR or CRTC2‐IDR mutants (left). Quantification of the size of droplets (right). e) Live‐cell imaging of ectopically expressed CRTC2‐eGFP in 293T cells. Arrows indicate representative CRTC2 puncta that fused over time. The dotted line area indicates the nucleus. f) Representative images of the FRAP experiment with ectopically expressed CRTC2‐eGFP in 293T cells (upper). The dotted line area indicates the nucleus. Quantification of FRAP data for CRTC2‐eGFP puncta (lower). g) Live‐cell images of ectopically expressed WT CRTC2‐eGFP or CRTC2‐IDR‐R>A mutant (R>A‐eGFP) in 293T cells (left). Quantification of cells with eGFP foci and western blot analysis of CRTC2‐eGFP or CRTC2‐IDR‐R>A expression (right). h) Live‐cell snapshots of ectopically expressed mCherry‐CRY2 fusion proteins fused with WT CRTC2‐IDR (upper) or CRTC2‐IDR‐R>A mutant (lower) in 293T cells before and after blue light stimulation (left). Quantification of cells with mCherry foci before and after blue light stimulation (right). Data are presented as means ± SEM. The unpaired two‐sided Student's *t*‐test was used for statistical analysis. ****p < 0.0001. n.s., not significant. Scale bar, 5 µm (a), 10 µm (b,d). All results are from more than three independent experiments.

cAMP signaling regulates gene expression by modulating the activity of downstream transcription effectors, including CREB, CREM, ATF1, and CRTCs. Interestingly, we observed that forskolin (FSK) treatment, which increased intracellular cAMP signal, induced both nuclear translocation and foci formation of CRTC2, but not other cAMP transcription effectors (**Figure** **1**a). Forming bright foci is frequently seen for proteins having an intrinsic capacity to undergo phase separation,^[^ ^1^, ^3^ ^]^ and we therefore wondered whether CRTC2 has this phase separation ability. To address this, we first analyzed the amino acid sequence of CRTC2 using algorithms that predict intrinsically disordered regions (IDRs) in proteins, which revealed that CRTC2 contains a large IDR in its C‐terminal region encompassing a transcription activation domain and a splicing domain (Figure S1a, Supporting Information).

There is accumulating evidence that purified IDRs of phase‐separating proteins tend to form liquid‐like droplets in vitro.^[^ ^46^ ^]^ We next investigated whether the IDR of CRTC2 forms such droplets. As shown in Figure S1b, Supporting Information, a solution containing purified recombinant CRTC2‐IDR‐eGFP turned opaque after addition of a crowding agent, 10% PEG 8000 (polyethylene glycol, molecular weight 8000), whereas an equivalent solution containing eGFP alone remained clear in the presence of the crowding agent. Fluorescence microscopy analysis revealed that purified CRTC2‐IDR‐eGFP protein could readily form droplets in the presence of 10% PEG 8000, and an increase of protein or crowding agent concentration resulted in an increase in the number and size of the droplets (Figure 1b; Figure S1c, Supporting Information). Furthermore, the droplets were readily dissolved when electrostatic interaction among CRTC2‐IDR‐eGFP molecules was inhibited by increasing the salt concentration in the solution (Figure S1d, Supporting Information); or when hydrophobic interaction was inhibited by adding 1,6‐hexanediol (Figure S1e, Supporting Information). These results indicate that both electrostatic and hydrophobic interaction are required for the formation of CRTC2‐IDR droplets. Fluorescence recovery after photobleaching (FRAP) analysis showed that CRTC2‐IDR‐eGFP droplets exhibited fast recovery after photobleaching (Figure 1c), indicating that CRTC2‐IDR molecules are highly mobile inside the droplets.

Amino acid composition in the CRTC2‐IDR region is highly conserved between human and mouse, especially for serine (S), tyrosine (Y), arginine (R), and lysine (K) residues (Figure S1f,g, Supporting Information). These amino acids are reportedly critical for phase separation of several proteins.^[^ ^47^, ^48^, ^49^, ^50^ ^]^ To identify the key amino acids in CRTC2‐IDR for phase separation, we generated a series of eGFP‐tagged CRTC2‐IDR mutants, in which all of the conserved S, Y, R, or K residues were replaced by alanine (A). We found that R>A mutation dramatically abolished the droplet formation of CRTC2‐IDR under conditions in which the wild‐type IDR readily formed droplets, whereas the other CRTC2‐IDR mutants (S>A, Y>A, and K>A) retained their abilities to form large droplets (Figure 1d), indicating that R residues are essential for CRTC2‐IDR phase separation in vitro.

As described above, FSK treatment induced focus formation of eGFP‐CRTC2 (Figure 1a), implying that CRTC2 might also undergo phase separation in vivo. Consistent with this, FSK‐induced eGFP‐CRTC2 droplets readily fused into larger structures over time (Figure 1e), indicating their liquid‐like nature. Furthermore, in vivo FRAP assays also revealed a quick recovery of eGFP‐CRTC2 fluorescence after photobleaching, on a time scale of seconds (Figure 1f), further suggesting that CRTC2 undergoes phase separation in vivo. In addition, this in vivo phase separation capacity of CRTC2 was abolished by systematic R>A mutation in the IDR (Figure 1g).

An optogenetic platform has recently been developed to test the phase separation ability of a protein of interest in living cells.^[^ ^51^ ^]^ *Arabidopsis thaliana* cryptochrome 2 (CRY2) is a light‐sensitive self‐associating protein that can be fused to a protein with phase separation capacity. So‐called optoDroplets of this fusion protein can be induced upon blue light exposure.^[^ ^51^ ^]^ To further test whether the CRTC2‐IDR could be induced to condense in living cells, we expressed CRY2‐mCherry‐tagged CRTC2‐IDR or CRTC2‐IDR‐R>A in 293T cells and performed optoDroplet assays. We found that blue light (488 nm laser) stimulation substantially increased the formation of optoDroplets in cells expressing CRTC2‐IDR‐CRY2‐mCherry (Movie S1, Supporting Information) but failed to do so in cells expressing CRTC2‐IDR‐R>A (Movie S2, Supporting Information) mutant protein (Figure 1h). These results indicate that CRTC2 can form phase‐separated condensates in living cells.

### 2.2 P‐TEFb Interacts with CRTC2 and Is Required for CRTC2 Transactivation

**Figure 2:**
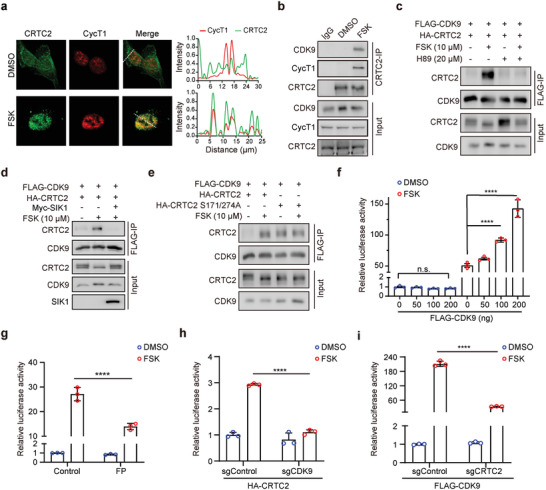
P‐TEFb interacts with CRTC2 and enhances CRTC2‐dependent transcription. a) Immunofluorescence analysis of the co‐localization of CRTC2 and CycT1 in HeLa cells treated with DMSO or FSK (left). Line scans along the dotted lines in the images (right). b) Co‐IP assay examining the interaction between endogenous CRTC2 and P‐TEFb in 293T cells treated with DMSO or FSK for 1 h. c) Co‐IP assay examining the interactions between FLAG‐CDK9 and HA‐CRTC2 in 293T cells treated with the indicated concentration of FSK or H89. d) Co‐IP assay examining the interactions between FLAG‐CDK9 and HA‐CRTC2 in 293T cells ectopically expressing Myc‐SIK1. e) Co‐IP assay examining the interactions between FLAG‐CDK9 and HA‐CRTC2 (WT or S171/274A mutants) in 293T cells. f) Quantification of CRE‐luc luciferase activity in 293T cells transfected with the indicated amounts of FLAG‐CDK9 plasmids and treated with DMSO or FSK (10 µm) for 6 h. g) Quantification of CRE‐luc luciferase activity in 293T cells treated with flavopiridol (FP, 300 nm) and FSK (10 µm) for 6 h. h) Quantification of CRE‐luc luciferase activity in HA‐CRTC2‐expressing 293T cells infected with lentivirus carrying sgRNA against CDK9 (sgCDK9) or GFP (sgControl) and treated with DMSO or FSK (10 µm) for 6 h. i) Quantification of CRE‐luc luciferase activity in FLAG‐CDK9‐expressing 293T cells infected with lentivirus carrying sgRNA against CRTC2 (sgCRTC2) or GFP (sgControl) and treated with DMSO or FSK (10 µm) for 6 h. Data are presented as means ± SEM. The unpaired two‐sided Student's *t*‐test was used for statistical analysis. ****p < 0.0001; n.s., not significant. All results are from more than three independent experiments.

We have previously reported that cAMP signaling can promote transcription elongation of cAMP‐responsive genes by liberating P‐TEFb from its inhibitory complex, which consists of 7SK snRNA, HEXIM1, LARP7, and MePCE.^[^ ^33^ ^]^ Recently, Lu et al. reported that the histidine‐rich domain of CycT1 promoted the formation of phase‐separated condensates of the P‐TEFb complex and targeted the PoI II CTD into this environment to ensure the hyperphosphorylation and efficient elongation of Pol II.^[^ ^52^ ^]^ We therefore asked whether cAMP‐induced CRTC2 condensates colocalize with P‐TEFb condensates. Immunofluorescence analysis revealed that cAMP induced the co‐localization of CRTC2 condensates with those of P‐TEFb in the nucleus (**Figure** **2**a). We then sought to determine whether CRTC2 interacts with P‐TEFb. Co‐immunoprecipitation analysis showed that endogenous CRTC2 associated with both CDK9 and CycT1 upon FSK treatment in 293T cells (Figure 2b).

CRTC2 can be phosphorylated by salt‐induced kinase 1 (SIK1), which prevents CRTC2 nuclear translocation.^[^ ^30^ ^]^ cAMP signaling induces CRTC2 dephosphorylation and translocation into the nucleus by PKA‐mediated phosphorylation and inactivation of SIK1.^[^ ^53^ ^]^ We wondered whether PKA activity is required for the cAMP‐induced interaction of CRTC2 and P‐TEFb. Pretreating the cells with H89, a PKA inhibitor, for 30 min completely blocked the CRTC2–CDK9 interaction (Figure 2c). In addition, this cAMP‐induced CRTC2–CDK9 interaction was also blocked by the presence of SIK1 activity (Figure 2d). Taken together, these results indicate that PKA activity is essential for cAMP‐induced association between CRTC2 and P‐TEFb.

To further characterize their interaction, we generated a CRTC2 mutant, S171/274A, which is unphosphorylated and constitutively active in the absence of FSK treatment. As shown in Figure 2e, in contrast to WT CRTC2, which interacted with CDK9 only in the presence of FSK, the CRTC2 S171/274A mutant was able to interact with CDK9 independent of FSK treatment. Collectively, these data indicate that cAMP signaling induces an interaction between CRTC2 and CDK9.

cAMP induces CRTC2 translocation into the nucleus and activation of CRTC2‐regulated gene expression. We then tested whether P‐TEFb is required for CRTC2 transcriptional activity. The cAMP signal can activate a luciferase reporter containing a cAMP‐responsive element (CRE), which requires CRTC2 activity. Thus, cAMP‐induced transactivation of CRTC2 can be assessed by this CRE‐luciferase reporter. We found that CDK9 dose‐dependently enhanced the cAMP‐induced CRE–luciferase reporter activity (Figure 2f); this cAMP‐induced reporter activation was repressed by treating cells with flavopiridol (FP), a CDK9 inhibitor (Figure 2g), indicating that CDK9 is required for the cAMP‐induced gene activation. Further, we observed that CDK9 depletion substantially repressed the CRTC2‐driven reporter activation (Figure 2h; Figure S2a, Supporting Information) and depletion of CRTC2 diminished the CDK9‐driven CRE activity in the presence of cAMP (Figure 2i; Figure S2b, Supporting Information). Taken together, these results indicate that the interaction with CDK9 is essential for cAMP‐induced CRTC2 transactivation.

### 2.3 CRTC2 Extracts P‐TEFb from the Inhibitory 7SK snRNP Complex

**Figure 3:**
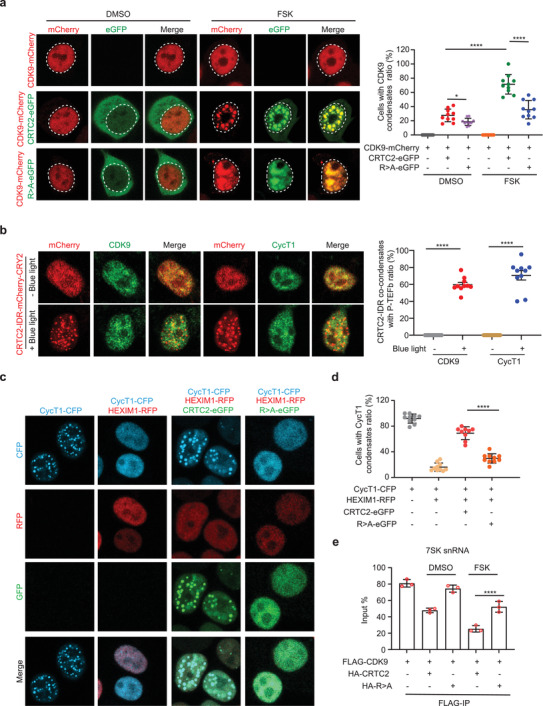
CRTC2 extracts P‐TEFb from HEXIM1‐containing inhibitory complex. a) Live‐cell imaging of 293T cells co‐expressing CDK9‐mCherry and CRTC2‐eGFP or R>A‐eGFP and treated with DMSO or FSK (10 µm) for 1 h (left). The dotted line area indicates the nucleus. Quantification of cells with CDK9‐mCherry foci upon treatment with DMSO or FSK (right). b) Immunofluorescence analysis of co‐condensates of CRTC2‐IDR‐mCherry‐CRY2 with CDK9 or CycT1 in 293T cells before or after blue light stimulation (left). Quantification of cells with CRTC2‐IDR co‐condensates with CDK9 or CycT1 (right). c) Live‐cell imaging of 293T cells co‐expressing CycT1‐CFP, HEXIM1‐RFP, CRTC2‐eGFP, and R>A‐eGFP as indicated. d) Quantification of cells containing CycT1‐CFP foci. e) RNA‐IP assay examining the association between FLAG‐CDK9 and 7SK snRNA in 293T cells co‐expressing HA‐CRTC2 or HA‐R>A and treated with DMSO or FSK. Data are presented as means ± SEM. The unpaired two‐sided Student's *t*‐test was used for statistical analysis. ****p < 0.0001. All results are from more than three independent experiments.

Our data indicate that cAMP induces formation of CRTC2 nuclear condensates as well as an interaction between P‐TEFb and CRTC2. Our next question was whether P‐TEFb was incorporated into these cAMP‐induced CRTC2 condensates. Although showing dispersed nuclear distribution when expressed alone, CDK9 was concentrated and incorporated into the CRTC2 nuclear condensates upon FSK treatment, whereas systematic R>A mutation in CRTC2 IDR abolished this CDK9 incorporation (**Figure** **3**a). To further characterize the co‐condensation between CRTC2 and P‐TEFb, we expressed CRTC2‐IDR‐CRY2‐mCherry in 293T cells and performed optoDroplet assays. Upon blue light stimulation, we observed that endogenous P‐TEFb was incorporated into CRTC2 condensates in the nucleus (Figure 3b).

The majority of P‐TEFb interacts with HEXIM1 and is sequestered in the inhibitory 7SK snRNP complex in unstimulated conditions.^[^ ^35^ ^]^ We next asked whether cAMP‐induced CRTC2 condensates could antagonize HEXIM1 and extract P‐TEFb from its inhibitory complex. Consistent with a recent report,^[^ ^54^ ^]^ we observed that CycT1 formed droplets when expressed alone, but they were dissolved by HEXIM1 co‐expression (Figure 3c). Notably, co‐expression of CRTC2 restored the ability of CycT1 to form droplets despite the presence of HEXIM1, indicating that CRTC2 is able to extract and concentrate CycT1 from the inhibitory complex, thereby activating P‐TEFb. In contrast, the CRTC2 R>A mutant, which lacks the ability to form condensates, could not extract and concentrate CycT1 from the inhibitory complex (Figure 3c,d). These results suggest that CRTC2 phase separation can help to free and activate P‐TEFb. In support of this, ectopic expression of WT CRTC2, but not the CRTC2 R>A mutant, disrupted the inhibitory complex, as shown by a reduced interaction of CDK9 with 7SK snRNA (Figure 3e). Collectively, these data indicate that cAMP‐induced CRTC2 phase separation can antagonize HEXIM1 and extract P‐TEFb from its inhibitory complex.

### 2.4 CRTC2 Forms Nuclear Condensates in Cyst‐Lining Epithelial Cells

**Figure 4:**
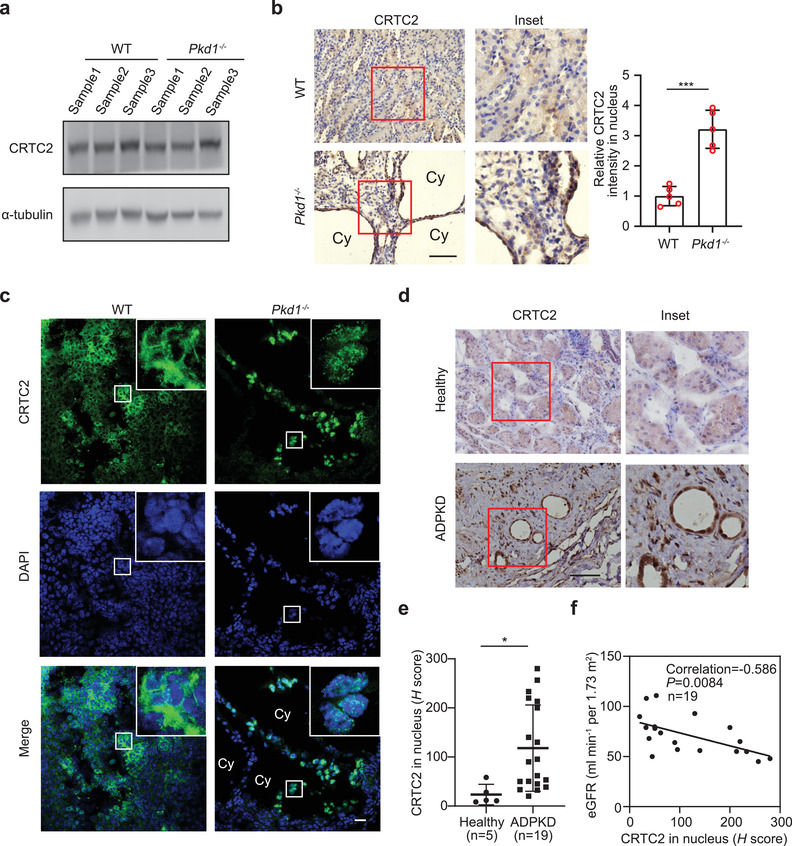
CRTC2 forms nuclear droplets in cystic epithelial cells. a) Western blot analysis of CRTC2 levels in kidneys from WT and *Pkd1^−/−^* mice (*n*  =  3). b) Representative immunohistochemistry (IHC) images of CRTC2 in kidneys of WT and *Pkd1^−/‐^* mice (left). Quantification of the signal intensity of nuclear CRTC2 (right) (*n*  =  5). Cy, large cyst. c) Representative IF images of CRTC2 in the kidney of WT and *Pkd1^−/‐^* mice. Cy, large cyst. d) Representative IHC images of CRTC2 in kidneys from healthy people (*n* = 5) and ADPKD patients (*n* = 19). e) Quantification of the signal density of nuclear CRTC2 of (d). f) Correlation between the signal intensity of nuclear CRTC2 and eGFR in patients with ADPKD. Pearson's correlation coefficients are displayed for each graph. *p* values for (f) were determined using linear regression analysis. Data are presented as means ± SEM. The unpaired two‐sided Student's *t*‐test was used for statistical analysis. ***p < 0.001, *p < 0.05. Scale bar, 20 µm (b–d).

Dysregulation of cAMP signaling is involved in the pathogenesis of many diseases, including ADPKD, a pathological condition marked by high levels of cAMP in renal epithelial cells.^[^ ^41^, ^43^ ^]^ To explore whether CRTC2 condensates are involved in the pathogenesis of cAMP‐dysregulated diseases, we sought to examine their involvement in ADPKD. Western blotting analysis revealed no evident difference in CRTC2 expression levels between normal and ADPKD mouse kidneys (**Figure** **4**a). Strikingly, immunohistochemical analysis revealed substantially distinct staining patterns for CRTC2 in normal and ADPKD mouse kidneys, with more intense nuclear staining in ADPKD kidneys than in normal kidneys (Figure 4b). In addition, immunofluorescence analysis also showed distinct CRTC2 localization patterns, with dispersed cytoplasmic staining in normal kidneys but clear nuclear staining with prominent punctate structures in the cyst‐lining cells in ADPKD mouse kidneys (Figure 4c). We also observed similar CRTC2 distribution patterns in normal human kidneys and kidneys from ADPKD patients (Figure 4d). Quantitative analysis of CRTC2 nuclear distribution by *H* score revealed a stronger nuclear intensity of CRTC2 in ADPKD kidneys than in normal kidneys (Figure 4e). Furthermore, the nuclear intensity of CRTC2 correlated negatively with the estimated glomerular filtration rate (eGFR) (Figure 4f), suggesting that CRTC2 nuclear accumulation is correlated with disease progression. Taken together, these data demonstrate that CRTC2 is aberrantly enriched in the nucleus and forms nuclear condensates in cystic kidneys.

### 2.5 Loss of CRTC2 Suppresses Cyst Formation in the ADPKD Mouse Model

**Figure 5:**
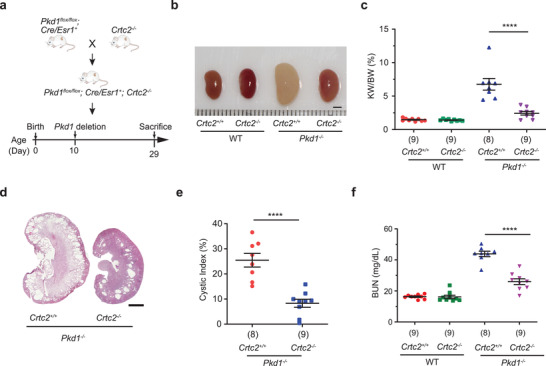
Loss of CRTC2 delays cyst formation in an ADPKD mouse model. a) Experimental design of the ADPKD mouse model. b) Representative kidneys from mice at P29 with indicated genotype. (*n* = 8 or 9 biologically independent mice per group). c) KW/BW ratios of the indicated groups of mice in the ADPKD model. The number below each bar refers to the number of samples analyzed. d) Hematoxylin and eosin (H&E) staining of kidney sections from mice at P29 with indicated genotype. (*n* = 8 or 9 biologically independent mice per group). e) Cystic index of H&E‐stained kidneys from mice at P29 with indicated genotype. The number below each bar refers to the number of samples analyzed. f) Plasma BUN levels of mice from the indicated groups. The number below each bar refers to the number of samples analyzed. Data are presented as means ± SEM. The unpaired two‐sided Student's *t*‐test was used for statistical analysis. ****p < 0.0001. Scale bar, 2 mm (b,d).

To further explore the role of CRTC2 in ADPKD, we investigated the effect of CRTC2 deletion on ADPKD progression. To do this, we crossed *Crtc2* knockout mice with an orthologous ADPKD mouse model, *Pkd1^flox/flox^; Cre/Esr1^+^* (*Pkd1^−/−^*) mice. *Pkd1* deletion was induced by tamoxifen injection at postnatal day 10 (P10) to initiate cystogenesis, and kidneys were collected at P29 for evaluation of disease progression (**Figure** **5**a). As shown in Figure 5b–f and Figure S3a,b, Supporting Information, deletion of *Crtc2* in the ADPKD setting caused decreases in kidney size, the ratio of kidney weight to body weight (KW/BW), cystic index, and blood urea nitrogen (BUN), suggesting that *Crtc2* deletion alleviates cystogenesis and improves kidney function. In addition, we performed H&E staining for kidneys from *Pkd1^+/+^; Crtc2^+/+^* and *Pkd1^+/+^; Crtc2^−/−^* mice. As shown in Figure S3c,d, Supporting Information, kidney size and anatomical structure were similar between *Pkd1^+/+^;Crtc2^+/+^* and *Pkd1^+/+^; Crtc2^−/−^*, suggesting that CRTC2 KO does not affect kidney structure.

### 2.6 CRTC2 Predominantly Binds to Transcriptionally Active Chromatin in ADPKD Cells

**Figure 6:**
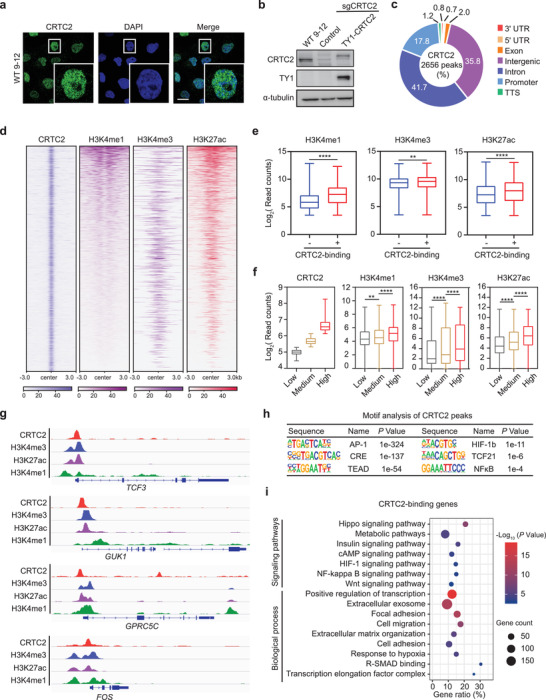
Genome‐wide CRTC2 localization in human ADPKD cells. a) Representative immunofluorescence images of endogenous CRTC2 in WT 9–12 cells. The result is from more than three independent experiments. b) Western blot analysis of whole‐cell lysates from WT 9–12 cells with indicated treatment. The result is from more than three independent experiments. c) Genomic distribution of CRTC2 in WT 9–12 cells. d) Heatmaps of normalized ChIP‐seq signals for CRTC2, H3K4me1, and H3K4me3, H3K27ac. The rows show 3 kb flanking the CRTC2 peak center. e) Boxplots of the normalized counts of H3K4me1, H3K4me3, and H3K27ac signals at CRTC2 binding + or − peaks. f) Boxplots of CRTC2, H3K4me1, H3K4me3, and H3K27ac reads in the indicated groups. Low: CRTC2 reads < 39; medium: CRTC2 reads 40–70; high: CRTC2 reads > 71. g) ChIP‐seq tracks of CRTC2, H3K4me3, H3K27ac, and H3K4me1 on representative genes. h) Motif analysis of CRTC2 peaks. i) GO and KEGG pathway enrichment analyses of CRTC2‐binding genes. Data are presented as means ± SEM. The unpaired two‐sided Student's *t*‐test was used for statistical analysis. ****p < 0.0001, **p < 0.01. Scale bar, 20 µm (a).

Translocation of CRTC2 into the nucleus triggers its activation and recruitment to cAMP‐responsive genes.^[^ ^32^ ^]^ To characterize the molecular mechanisms by which CRTC2 promotes cystogenesis in ADPKD, we first analyzed the genome‐wide distribution of CRTC2 by chromatin immunoprecipitation coupled with sequencing (ChIP‐seq) in WT 9–12 human ADPKD cells. We began by examining the expression and distribution of CRTC2 in this ADPKD cell model. As shown in **Figure** **6**a, staining for endogenous CRTC2 revealed discrete nuclear puncta, suggesting that CRTC2 forms condensates in ADPKD cells. Unfortunately, ChIP of endogenous CRTC2 using commercially available CRTC2 antibodies was unsuccessful; we therefore replaced endogenous CRTC2 with TY1‐tagged CRTC2 to generate an engineered WT 9–12 cell line, in which CRTC2 expression is comparable to the parental cells (Figure 6b). We then performed ChIP‐seq in these engineered cells to profile the genome‐wide occupancy of TY1‐CRTC2 using a TY1 antibody. In this way, 2656 genomic regions were identified as TY1‐CRTC2 binding sites (Figure 6c). We profiled the landscape of transcriptionally active chromatin regions by performing ChIP‐seq analyses of H3K4me1, H3K4me3, and H3K27ac, histone modifications that are associated with active transcription. Integrative analysis of ChIP‐seq data revealed that CRTC2 binding sites are enriched for these active chromatin marks (Figure 6d). In addition, CRTC2‐bound genomic regions displayed a markedly higher density of H3K4me1, H3K4me3 and H3K27ac modifications compared with the average signal at regions without CRTC2 binding (Figure 6e). The enrichment of all three modifications increased gradually as CRTC2 binding increased (Figure 6f). Representative ChIP‐seq profiles are shown in Figure 6g. Motif analysis of CRTC2‐binding peaks revealed that CRTC2 enriched in DNA‐binding motifs for CREB and other reported ADPKD‐associated transcriptional factors, including AP‐1,^[^ ^55^ ^]^ TEAD,^[^ ^56^, ^57^ ^]^ HIF‐1b,^[^ ^58^ ^]^ and NF‐*κ*B^[^ ^59^ ^]^ (Figure 6h). Consistently, functional enrichment analysis showed that CRTC2‐binding genes were enriched in multiple ADPKD‐associated pathways, including Hippo signaling, metabolic pathway, cAMP signaling, HIF‐1 signaling, and NF‐*κ*B signaling pathway. Gene ontology analysis identified the enriched biological processes, such as positive regulation of transcription, extracellular exosome, and focal adhesion (Figure 6i).

### 2.7 CRTC2 Activates Cystogenesis‐Associated Genes in ADPKD

**Figure 7:**
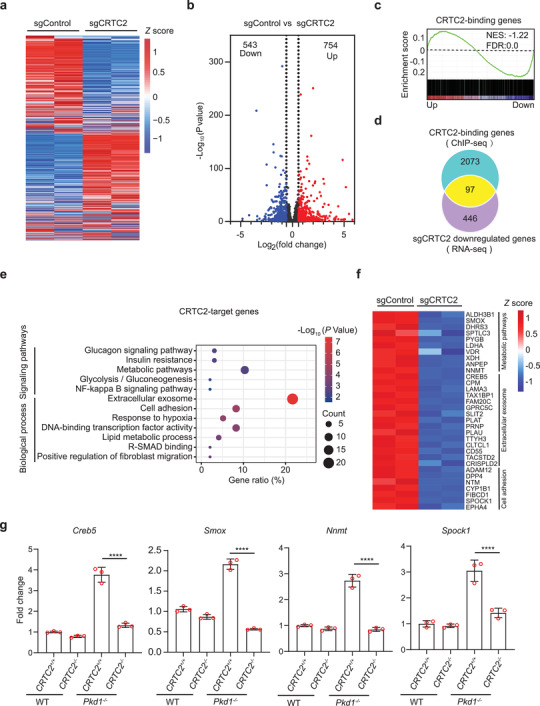
CRTC2 regulates the expression of cystogenesis‐associated genes in human ADPKD cells. a) Heatmap of gene expression in WT 9–12 cells transfected with sgControl or sgCRTC2. Rows show the *Z* scores calculated for each group. b) Volcano plots showing differentially expressed genes. c) Gene set enrichment analysis displaying CRTC2‐binding gene set enriched for genes downregulated in sgCRTC2 cells. NES, normalized enrichment score. d) Venn diagram showing the overlap of sgCRTC2 downregulated genes and CRTC2‐binding genes, referred to as CRTC2‐target genes. e) GO and KEGG pathway enrichment analyses of CRTC2‐target genes. f) Heatmap of expression values of genes enriched in the indicated processes. Rows show the *Z* scores calculated for each group. g) RT‐qPCR analysis of mRNA levels of representative CRTC2‐target genes in kidneys from the indicated mouse groups. Data are presented as means ± SEM. The unpaired Student's two‐sided *t*‐test was used for statistical analysis. ****p < 0.0001. These results are from three independent experiments.

To examine the effect of CRTC2 activation on transcriptional output in ADPKD cells, we performed transcriptomic analyses by RNA sequencing (RNA‐seq) in WT 9–12 (Control) and WT 9–12 CRTC2 knockout (CRTC2 KO) cells. Transcriptomic profiles of CRTC2 KO cells differed substantially from those of Control cells (**Figure** **7**a), with 543 genes downregulated and 754 genes upregulated over 1.5‐fold in CRTC2 KO cells compared to Control cells (Figure 7b). We found that the average expression level of CRTC2‐binding genes was much higher than that of CRTC2‐nonbinding genes (Figure S3, Supporting Information). Gene set enrichment analysis revealed that the CRTC2‐binding gene set was enriched for genes downregulated in CRTC2 KO cells (Figure 7c). This is consistent with earlier reports that CRTC2 generally functions as a transcriptional activator. We found that 97 of the 543 downregulated genes had CRTC2 occupancy (Figure 7d), indicating that their expression is directly regulated by CRTC2; we name these genes CRTC2‐target genes. Pathway enrichment analysis revealed a remarkable enrichment for genes involved in cystogenesis‐associated pathways, such as metabolic pathways, cell adhesion, and extracellular exosome, as well as some known CRTC2‐regulated pathways, including insulin resistance and glucagon signaling pathway, which have also been reported to be perturbed in ADPKD^[^ ^60^, ^61^ ^]^ (Figure 7e,f). We next examined the expression of several CRTC2‐target genes in mouse kidneys from normal and ADPKD mice with or without *Crtc2* knockout (*Pkd1* ^+/+^ *Crtc2* ^+/+^, *Pkd1* ^+/+^ *Crtc2* ^−/−^, *Pkd1* ^−/−^ *Crtc2* ^+/+^, and *Pkd1* ^−/−^ *Crtc2* ^−/−^ mice) by performing RT‐qPCR assays. As shown in Figure 7g, the expression of these genes was elevated in ADPKD tissues and *Crtc2* knockout brought them down to near‐normal levels. Collectively, these results indicate that CRTC2 is essential for the activation of cystogenesis‐associated genes in ADPKD cells.

### 2.8 CRTC2 Condensates Facilitate P‐TEFb Recruitment and the Release of Paused Pol II on Cystogenesis‐Associated Genes

**Figure 8:**
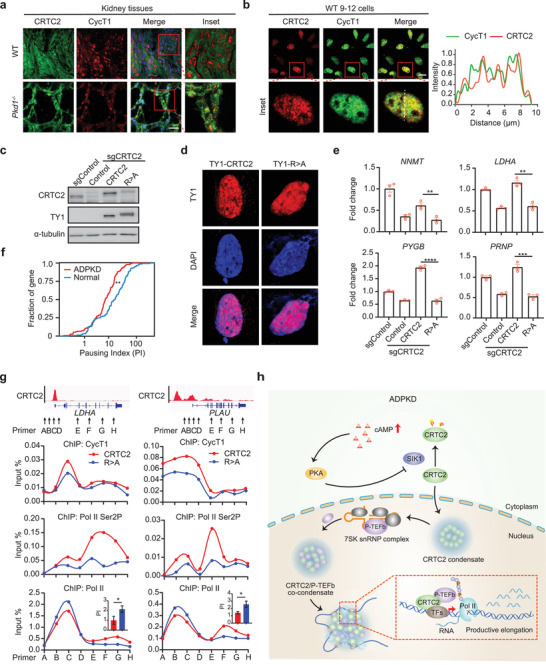
CRTC2 activates cystogenesis‐associated genes by promoting paused Pol II release. a) Immunofluorescence analysis of CRTC2 and CycT1 co‐localization in the kidneys of WT and *Pkd1^−/‐^* mice (*n* = 3). b) Immunofluorescence analysis of CRTC2 and CycT1 co‐localization in WT 9–12 cells (left). Line scan of the dashed line in the merged inset image (right). c) Western blot analysis of CRTC2 expression in WT 9–12 cells transfected with the indicated sgRNAs and constructs. d) Immunofluorescence analysis of ectopically expressed TY1‐CRTC2 and TY1‐CRTC2‐R>A in WT 9–12 cells with endogenous CRTC2 knocked out. e) RT‐qPCR analysis of representative CRTC2‐target genes in WT 9–12 cells transfected with the indicated sgRNAs and constructs. f) Cumulative curve of Pol II pausing index (PI) for CRTC2 target genes in normal and ADPKD kidney tissues. g) ChIP‐qPCR analysis of CycT1, Pol II Ser2P, and Pol II occupancy on *LDHA* and *PLAU* genes in WT 9–12 cells with endogenous CRTC2 replaced by TY1‐CRTC2 or TY1‐CRTC2‐R>A mutant. The inset bar graphs indicate the Pol II pausing index for *LDHA* and *PLAU* genes, respectively. h) Schematic illustration of how CRTC2 triggers the productive elongation of cystogenesis‐associated genes, ultimately leading to ADPKD progression. Data are presented as means ± SEM. The unpaired two‐sided Student's *t*‐test was used for statistical analysis. *p < 0.05, **p < 0.01, ***p < 0.001, ****p < 0.0001. Scale bar, 20 µm (a,b). All results are from more than three independent experiments.

Next, we investigated how CRTC2 activated the cystogenesis‐associated genes in ADPKD cells. We demonstrated above that CRTC2 condensates activate P‐TEFb by extracting it from 7SK‐snRNP inhibitory complex upon activation of cAMP signaling. We speculated that CRTC2 activates cystogenesis‐associated genes by promoting P‐TEFb‐mediated transcription elongation in ADPKD cells. To test this, we first examined whether P‐TEFb components were incorporated into CRTC2 condensates in ADPKD cells. We found that while CRTC2 and CycT1 displayed distinct cellular distribution in WT kidneys, they colocalized in the nucleus and exhibited discrete puncta staining in ADPKD kidneys (**Figure** **8**a). Consistent with this, we also observed colocalization of CRTC2 and CycT1 in nuclear condensates in WT 9–12 cells (Figure 8b). These results support the idea that CRTC2 condensates incorporate P‐TEFb in ADPKD cells.

We then wondered whether the phase separation capability of CRTC2 was required for the expression of CRTC2‐target genes. To test this, we analyzed the effect of disrupting CRTC2 condensates on the expression of CRTC2‐targets. CRTC2 condensates were disrupted by knocking out endogenous CRTC2, followed by transfection of a CRTC2 R>A mutant construct tagged with TY1. Western blotting revealed comparable levels of TY1‐CRTC2‐R>A mutant or TY1‐WT‐CRTC2 expression in these cells to that of endogenous CRTC2 in parental WT 9–12 cells (Figure 8c). Immunofluorescence analysis in these cells showed that TY1‐WT‐CRTC2 exhibited nuclear puncta staining, whereas the TY1‐CRTC2‐R>A mutant showed diffuse staining, supporting the idea that R>A mutation in CRTC2 disrupts its ability to form condensates in ADPKD cells (Figure 8d). We next performed RT‐qPCR analyses for several CRTC2‐target genes, including *NNMT*, *LDHA*, *PYGB*, and *PRNP*. As shown in Figure 8e, their expression was decreased in CRTC2‐depleted cells, and recovered in cells complemented with TY1‐WT‐CRTC2 but not with TY1‐CRTC2‐R>A mutant protein. These results indicate that the phase separation capacity of CRTC2 is required for the expression of CRTC2‐target genes.

We have previously reported that cAMP‐activated P‐TEFb promotes transcription elongation of cystogenesis‐associated genes by enhancing the release of paused Pol II at gene promoter‐proximal sites in ADPKD kidneys.^[^ ^33^ ^]^ We then asked whether CRTC2 regulates Pol II pausing release in ADPKD cells. We calculated a Pol II pausing index for CRTC2‐target genes in normal kidney and ADPKD kidney by re‐analyzing the Pol II ChIP‐seq data from our earlier study.^[^ ^33^ ^]^ The pausing index was determined by comparing Pol II enrichment on gene promoter‐proximal regions with that on the corresponding gene bodies. We found that CRTC2‐target genes had a lower pausing index in kidney from ADPKD mouse than in normal kidney (Figure 8f), suggesting that CRTC2 indeed promotes Pol II pausing release at these genes.

To test whether phase separation of CRTC2 affects the pausing release on CRTC2‐target genes, we then examined the impact of disrupting CRTC2 condensates on P‐TEFb recruitment and Pol II elongation on these genes. As shown in Figure 8g CycT1 recruitment on two representative genes (*LDHA* and *PLAU*) was markedly lower in cells expressing TY1‐CRTC2‐R>A mutant than in cells with WT CRTC2. The enrichment of elongating Pol II, characterized by Ser2 phosphorylation (Ser2P) on Pol II CTD, on gene bodies also decreased after WT CRTC2 expression was replaced by TY1‐CRTC2‐R>A mutant expression (Figure 8g). Consistent with this, Pol II distribution on gene bodies decreased, but increased on promoter‐proximal regions in cells expressing TY1‐CRTC2‐R>A mutant, resulting in a higher pausing index. Overall our findings indicate that CRTC2, by compartmentalizing and activating P‐TEFb, triggers the productive elongation of cystogenesis‐associated genes, ultimately leading to ADPKD progression.

## 3 Discussion

Phase separation is implicated in both signal transduction and regulation of gene expression.^[^ ^4^, ^5^, ^6^ ^]^ Here we report that phase separation of CRTC2 is important for coupling cAMP signal transduction to transcription elongation. Both perturbation of signaling pathways and dysregulation of gene expression contribute to the transformation of normal cells into diseased cells. We reveal here that dysregulation of cAMP signaling results in pathogenic phase separation of CRTC2 in ADPKD cells. Conversely, depletion of CRTC2 ameliorates ADPKD cystogenesis. Our findings provide evidence that the abnormal phase separation serves as an important bridge connecting signaling pathway disturbance to the dysregulation of gene expression.

Transcription elongation is an important regulatory step in gene expression,^[^ ^62^, ^63^ ^]^ and the P‐TEFb complex is the most powerful regulator of transcription elongation.^[^ ^64^ ^]^ In a previous work, we found that P‐TEFb is activated by cAMP signaling and is involved in cystogenesis‐associated gene expression, but how exactly the activated P‐TEFb interacts with transcriptional regulators downstream of cAMP signaling, and how it achieves gene‐specific activation on the cystogenesis‐associated genes, remained obscure. In this study, we demonstrate that P‐TEFb is incorporated into cAMP‐induced CRTC2 phase‐separated condensates, thereby enhancing the recruitment of P‐TEFb and CRTC2 to cAMP‐responsive genes and cooperatively promoting their expression. These findings provide a mechanism by which P‐TEFb, as a general regulator of transcription elongation, achieves signal‐dependent selectivity of gene activation (Figure 8h).

Recent studies have implicated phase separation in many diseases, such as neurodegenerative diseases and cancers.^[^ ^7^, ^8^, ^9^, ^10^ ^]^ We previously show that transcription factor NRF2 can form phase‐separated condensates to activate antioxidant genes in normal kidney. In contrast, NRF2 is downregulated and NRF2 condensates are lost in ADPKD.^[^ ^65^ ^]^ In the current study, we find that CRTC2 translocates to nucleus and forms condensates in cystic cells, which now provides evidence that abnormal phase separation also occurs in ADPKD cells, and this first report for a renal disease further expands the spectrum of diseases associated with abnormal phase separation. ADPKD is a serious threat to human health, with more than half of the patients with ADPKD developing kidney failure before the age of 60.^[^ ^66^ ^]^ Our data demonstrate that genetic depletion of CRTC2 greatly reduces cystogenesis. These findings prompt us to speculate that CRTC2 will be an effective therapeutic target specific to ADPKD cystogenesis. Since the cAMP signaling pathway is also crucial for maintaining homeostasis in normal cells, direct blocking of the cAMP signaling pathway might well cause damage to normal cells, resulting in serious side effects. CRTC2 phase‐separated condensates occur specifically in ADPKD cystic epithelial cells, and not in normal cells. Abnormally activated cAMP in ADPKD cells induces abundant accumulation of CRTC2 in the nucleus, wherein CRTC2 crosses the threshold concentration for phase separation. Thus, interventions specifically targeting the formation of CRTC2 phase‐separated condensates should be excellent candidates for achieving specific destruction of signal transduction in diseased cells, while avoiding damage to normal cells. Recent studies indicate that several small molecules that specifically target the formation of phase‐separated condensates hold promise in disease intervention.^[^ ^67^, ^68^ ^]^ Searching for inhibitors targeting the CRTC2 condensates may therefore be useful for developing new therapeutics for ADPKD.

## 4 Experimental Section

### Cell Culture, Transfection, and Infection

293T and HeLa cells were cultivated in Dulbecco's Modified Eagle Medium (Biological Industries) supplemented with 10% fetal bovine serum (FBS, Biological Industries) and 1% penicillin‐streptomycin (GIBCO). WT 9–12 cells were cultured in Dulbecco's modified Eagle's medium/F‐12 medium (Biological Industries) supplemented with 10% fetal bovine serum (FBS) (Biological Industries) and 1% penicillin‐streptomycin (GIBCO). Cell transfection was performed using Lipofectamine 3000 (Thermo Scientific) or polyethyleneimine (PolyScience) according to the manufacturer's instructions. The CRISPR–Cas9 system was used to knockout CRTC2 in WT 9–12 cells. The stable sgCRTC2 cells expressing TY1‐WT‐CRTC2 or TY1‐CRTC2‐R>A were generated by infecting with lentivirus.

### Plasmids

The CRTC2‐eGFP, CREB1‐eGFP, ATF1‐eGFP, CREM‐eGFP, and CRTC2 mutation R>A‐eGFP constructs were generated by PCR and sub‐cloned into the pEGFP‐C1 vector. CRTC2‐IDR‐eGFP were generated by PCR and sub‐cloned into the pGEX vector. Mutations of CRTC2‐IDR (S>A, Y>A, K>A, and R>A) were synthesized by GENEWIZ and similarly cloned into the pGEX vector. CRTC2‐IDR‐mCherry‐CRY2 were generated by PCR and sub‐cloned into pHR‐mCh‐CRY2 WT vector. FLAG‐CDK9, HA‐CRTC2, Myc‐SIK1, HA‐CRTC2 S171/274A, and HA‐CRTC2‐R>A were generated by PCR and sub‐cloned into the pcDNA5 vector. CycT1‐CFP, HEXIM1‐mRFP, and CDK9‐mCherry were provided by Chengqi Lin (Southeast University, Nanjing, China).

### Recombinant Protein Purification

GST‐eGFP‐tagged CRTC2‐IDR and mutant proteins were expressed and purified from BL21 competent *Escherichia coli*. pGEX expression plasmids containing GST tag and CRTC2‐IDR with eGFP were transformed into BL21 cells. After induction with 200 µm isopropyl *β*‐D‐1‐thiogalactopyranoside (IPTG) overnight at 16 °C, cells were lysed and sonicated in buffer (50 mm Tris‐HCl, 100 mm NaCl, 0.2 mm phenylmethylsulfonyl fluoride (PMSF), 1 mm dithiothreitol (DTT), 1% Triton X‐100, and containing protease inhibitor cocktail). Then lysates were cleared at 15 000 rpm at 4 °C for 20 min. The supernatants were incubated with Glutathione‐Sepharose 4B beads (GE Healthcare Biosciences A, 17‐0756‐05) for overnight at 4 °C. Then the beads were washed with high salt (50 mm Tris‐HCl, 500 mm NaCl) and low salt (50 mm Tris‐HCl, 100 mm NaCl) buffer. Proteins were eluted with HRV 3C Protease (Thermo Scientific, 88 946) for 20 h at 4 °C.

### Droplet‐Formation Assay

Purified proteins were diluted to varying concentrations in buffer containing 20 mm Tris‐HCl pH 7.5 and 1 mm DTT with the indicated salt concentrations. Protein solution (5 µL) was loaded onto a glass slide, covered with a coverslip and imaged using fluorescence microscope (Leica). The sizes of the droplets were quantified using ImageJ.

### Live Cell Imaging

293T cells were seeded on glass‐bottom cell culture dishes (NEST) and transfected with indicated plasmids. Twenty‐four hours after transfection, the cells were treatment with Forskolin (FSK, 10 µm, Sigma) for 1 h or not. Fluorescence images were acquired on an inverted ZEISS LSM900 confocal microscope with a 63×, 1.4 NA oil immersion objective lens using LAS V4.4 software and a CCD camera. The 488 nm laser was used for image of eGFP‐fusion proteins, 430 nm laser was used for excitation of CFP‐fusion proteins, while the 555 nm laser was used for image the mRFP or mCherry fusion proteins. Images were acquired at intervals of indicated times and were analyzed with ImageJ to identify fusion events.

### FRAP and Data Analysis

293T cells expressing CRTC2‐eGFP were treated with FSK, and FRAP experiments were done on nucleus CRTC2 condensates using the bleaching mode in Zen. The region of interest was selected on either the entire condensate or part of it using a circle of ≈1 µm × 1 µm in size. Bleaching was done with a 488 nm Argon laser at 100% power. Two and 20 rounds of imaging were performed before and after bleaching until fluorescence signal recovery, respectively.

### OptoDroplet‐Related Assays

293T cells were seeded on glass‐bottom cell culture dishes (NEST) and IDR‐mCherry‐CRY2 or R>A mutant plasmids were transfected into the cells. One day after transfection, images were acquired on ZEISS LSM900 confocal microscope with a 63×, 1.4 NA oil immersion objective lens using ZEN 3.0 software and a CCD camera. To activate the self‐association of CRY2, cells were excited with a 488 nm laser for 6 s every 6 s, during which the mCherry signal was recorded. To analyze the co‐condensation between CRTC2‐IDR and P‐TEFb, IDR‐mCherry‐CRY2 plasmids were transfected into 293T cells. One day after transfection, cells were excited with a 488 nm laser for 5 min. Then incubated with primary antibody against CDK9 (Santa Cruz, sc‐13130) or CycT1 (Santa Cruz, sc‐8127). The samples were imaged by ZEISS LSM900 confocal microscope with a 63×, 1.4 NA oil immersion objective lens using LAS V4.4 software and a CCD camera.

### Co‐IP and RNA‐IP

Co‐IP assay were performed as described previously.^[^ ^69^ ^]^ Cells were lysed with NP‐40 lysis buffer (150 mm NaCl, 1.0% NP‐40, 50 mm Tris‐HCl pH 8.0, containing protease inhibitor cocktail). Corresponding antibodies were added into tissue lysates and incubated overnight at 4 °C. Proteins were immunoprecipitated by Dynabeads Protein G (Invitrogen, 10003D) overnight at 4 °C. Then the authors washed the beads with NP‐40 lysis buffer 3 times. For Co‐IP assay, the beads were boiled for 10 min for Western blotting analysis. For RNA‐IP assay, RNA was isolated from the beads using TRIzol (Invitrogen, 15 596 018) for qRT‐PCR analysis. 7SK snRNA primers are provided in Table S1, Supporting Information.

### ChIP‐seq Analysis

ChIP assays were performed mainly as previously described.^[^ ^70^ ^]^ WT 9–12 cells were cross‐linked with 1% formaldehyde for 10 min at room temperature (RT). Then cross‐linking was stopped by 150 mm glycine at RT for 5 min. Cells were washed with cold PBS and collected using ChIP lysis buffer (1% SDS, 10 mm EDTA, 50 mm Tris‐HCl pH 8.0, and containing protease inhibitor cocktail). Cells were fragmented to a size range of 500–1000 base pairs with a Bioruptor Sonicator. Immunoprecipitation was performed using 2 µg TY1 antibody (Invitrogen, MA5‐23513) and Dynabeads Protein G (Invitrogen, 10003D). After washing, the Dynabeads were eluted by elution buffer (1% sodium dodecyl sulfate, 0.1 m NaHCO_3_) and reversal cross‐linking in 65 °C overnight. After elution and reversal cross‐linking, DNA was purified and sequenced on the Illumina NovaSeq 6000 platform. Quality control was performed using FastQC v0.11.8 software. Clean reads were mapped to the reference genome (hg38) using Bowtie2 v2.3.4.1. ChIP‐seq peaks were detected by the peak‐finding algorithm MACS (model‐based analysis for ChIP‐seq, version MACS‐1.4.2) software. Bam files were converted to bigwig files by Deeptools (v3.4.3).

### ChIP‐qPCR Analysis

ChIP experiments were performed following the procedures described in the above ChIP‐seq analysis section. For Pol II and Pol II Ser2P ChIP, immunoprecipitation was performed using 2 µg Pol II antibody (Cell Signaling Technology, 14 958) or Pol II Ser2P antibody (Cell Signaling Technology, 13 499). For CycT1 ChIP, cells were double crosslinked by incubation with DMA (Sangon Biotech) for 1 h followed by treatment with 1% formaldehyde for 10 min at RT. Immunoprecipitation was performed using 2 µg CycT1 antibody (Santa Cruz, sc‐10750X). DNA was purified by extraction with phenol:chloroform and analyzed by RT‐qPCR analysis. Gene‐specific primers are provided in Table S2, Supporting Information.

### RNA‐seq Analysis

Total RNA was isolated from cultured cells using TRIzol (Invitrogen, 15 596 018) according to the manufacturer instructions and then sequencing by the Illumina NovaSeq 6000 platform. Low‐quality reads were filtered using internal software SOAPnuke v1.5.2. Clean reads were mapped to reference transcripts using Bowtie2 v2.2.5. Differentially expressed genes were calculated by DESeq2 algorithms. Fold changes of ≤ −1.5 or ≥ 1.5 and *p* < 0.05 were considered significantly differentially expressed. Function enrichment analysis was performed using KOBAS 3.0.

### RT‐qPCR

Total RNA was isolated from whole kidneys or cells using TRIzol (Invitrogen, 15 596 018). After RNA isolation, RNA was reverse‐transcribed by the cDNA Synthesis Kit (Roche). Gene‐specific primers are provided in Table S1, Supporting Information.

### Immunoblotting

Cells and tissue samples were lysed in RIPA lysis buffer (150 mm NaCl, 1.0% NP‐40, 50 mm Tris‐HCl pH 8.0, 1% sodium dodecyl sulfate, 0.5% sodium deoxycholate) with protease inhibitor cocktail (Roche). After incubation at 4 °C for 30 min, cell or tissue lysates were cleared and denatured in an SDS protein loading buffer. Protein samples were separated by SDS–PAGE, transferred to nitrocellulose membranes and immunoblotted with primary antibodies against CRTC2 (Proteintech, 12497‐1‐AP, dilution 1:1000), CDK9 (Santa Cruz, sc‐13130, dilution 1:1000), HEXIM1 (Proteintech, 15676‐1‐AP, dilution 1:5000), HA (Cell Signaling Technology, 3724, dilution 1:1000), FLAG (Sigma, A8592, dilution 1:5000), Myc (Cell Signaling Technology, 2278, dilution 1:2000), *α*‐tubulin (Proteintech, 11224‐1‐AP, dilution 1:5000), and TY1 (Invitrogen, MA5‐23513, dilution 1:2000). Secondary antibodies were goat anti‐rabbit IgG (H+L), horseradish peroxidase (HRP) (Invitrogen, 31 460), goat anti‐rat IgG (H+L), HRP conjugate (Proteintech, SA00001‐15), and goat anti‐mouse IgG (H+L), HRP (Invitrogen, 31 430).

### Immunohistochemistry

The human ADPKD tissues and mouse kidney tissues were perfused with 10% formalin overnight and paraffin‐embedded. Paraffin‐embedded tissues were cut into 7 µm sections and then treated with 3% H_2_O_2_ for 15 min and blocked by 5% BSA for 1 h at RT. Kidney sections were incubated with primary antibodies against CRTC2 (Proteintech, 12497‐1‐AP, dilution 1:100). After incubation with primary antibodies, sections were incubated with anti‐mouse/rabbit HRP secondary antibody (ZSGB‐BIO, pv‐6000). Assessment of the histopathology was evaluated in a blinded manner by two experienced pathologists. For *H* score calculation, nucleus staining intensity (0, 1+, 2+, or 3+) is determined for each cell in a fixed field. Then the percentage of cells at each staining intensity level is calculated. CRTC2 in nucleus *H* score is assigned using the following formula: (1 × (% cells 1+) + 2 × (% cells 2+) + 3 × (% cells 3+)).

### Immunofluorescence

Kidney sections or cells were blocked by 5% BSA and incubated with primary antibody against CRTC2 (Proteintech, 12497‐1‐AP, dilution 1:100) and CycT1 (Santa Cruz, sc‐8127, dilution 1:100). Then, sections or cells were incubated with goat anti‐mouse Alexa Fluor 488 and anti‐goat Alexa Fluor 555 secondary antibody (Thermo Fisher Scientific). The samples were imaged by fluorescence microscope (FV1000, Olympus).

### Mice Treatment

*Crtc2^−/−^* mice and *Pkd1^flox/flox^; Cre/Esr1^+^* mice were described previously.^[^ ^71^, ^72^ ^]^ The authors generated *Pkd1^flox/flox^; Crtc2^−/−^* mice by crossing *Pkd1^flox/flox^; Cre/Esr1^+^* mice with *Crtc2^−/−^* mice. The activity of Cre recombinase was induced by intraperitoneal injection of tamoxifen (Sigma, T5648) at P10 (10 mg per kg [body weight]) to generate ADPKD mouse model. The kidney tissues were collected on P29. Total area and cyst area measurement were evaluated using the National Institutes of Health ImageJ software. The cyst index was calculated as the ratio of cyst area to total area. Blood urea nitrogen (BUN) was measured in plasma using the QuantiChrom Urea Assay Kit (DIUR, BioAssay Systems). All mice care and experimental protocols were approved by the Ethical Committee of Tianjin Medical University (permit number: SYXK 2009‐00001).

### Human Kidney Specimens

Patient kidney specimens were obtained from cyst‐reduction surgery or nephrectomy. All participants involved provided informed consent. Human studies were approved by the Ethics Committee of Shandong Provincial Hospital (permit number: SWYX:NO. 2020‐013). A total of 19 archived paraffin‐embedded ADPKD kidney samples were used in the current study.

### Luciferase Assay

A total of 500 ng DNA (including 100 ng of CRE‐luciferase reporter construct and the indicated plasmids) was transfected into 293T cells using polyethyleneimine (PolyScience) according to the manufacturer's instructions. Eighteen hours later after transfection, the cells were treated with FSK or FP for 6 h. The luciferase activity was measured using Dual‐Luciferase Reporter Assay System (Promega).

### Statistical Analysis

GraphPad Prism 8.0 (GraphPad, USA) and PASW Statistics 18 (IBM, USA) were used for statistical analysis. Results were presented as means ± SEM for at least three independent experiments. All inclusion/exclusion criteria were preestablished, and no samples or animals were excluded from analyses. Statistical comparisons of two samples were performed using the unpaired two‐tailed Student's *t* test. Pearson's correlation coefficients were determined using linear regression analysis. All graphs were considered statistically significant when *p < 0.05, **p < 0.01, ***p < 0.001, ****p < 0.0001.

## Conflict of Interest

The authors declare no conflict of interest.

## Author Contributions

Z.M. and Y.S. contributed equally to this work. Z.M. and Y.S. performed biochemistry, cell biology, and animal studies, and wrote the manuscript. J.W. performed animal studies. Z.L. and X.C. performed bioinformatics analysis. L.D. performed biochemistry studies. Y.L. performed animal studies. Y.S. provided expertise on animal studies. H.X. provided human ADPKD samples. L.Z. analyzed the data and wrote the manuscript. Y.C. conceived, designed, and supervised the project, analyzed data, and wrote the manuscript.

## Supporting information

Supporting Information

Supplemental Movie 1

Supplemental Movie 2

## Data Availability

The data that support the findings of this study are openly available in the Gene Expression Omnibus database, reference number GSE173695.
