# Supplementary material for: cAMP‐Induced Nuclear Condensation of CRTC2 Promotes Transcription Elongation and Cystogenesis in Autosomal Dominant Polycystic Kidney Disease
Source: Adv Sci (Weinh). 2022 Jan 17;9(10):2104578. doi: 10.1002/advs.202104578 (PMC8981427; doi:10.1002/advs.202104578)
Supplement: Supplementary file 1 — Supporting Information [file ADVS-9-2104578-s002.pdf]

ADVANCED SCIENCE
Open Access

# Supporting Information

for *Adv. Sci.*, DOI 10.1002/advs.202104578

cAMP-Induced Nuclear Condensation of CRTC2 Promotes Transcription Elongation and Cystogenesis in Autosomal Dominant Polycystic Kidney Disease

*Zeyun Mi, Yandong Song, Jiuchen Wang, Zhiheng Liu, Xinyi Cao, Lin Dang, Yumei Lu, Yongzhan Sun, Hui Xiong, Lirong Zhang and Yupeng Chen**

ADVANCED SCIENCE
Open Access

# Supporting Information

for *Adv. Sci.,* DOI: 10.1002/advs.202104578

# cAMP-Induced Nuclear Condensation of CRTC2 Promotes Transcription Elongation and Cystogenesis in Autosomal Dominant Polycystic Kidney Disease

*Zeyun Mi, Yandong Song, Jiuchen Wang, Zhiheng Liu, Xinyi Cao, Lin Dang, Yumei Lu, Yongzhan Sun, Hui Xiong, Lirong Zhang, and Yupeng Chen**

## Figure S1

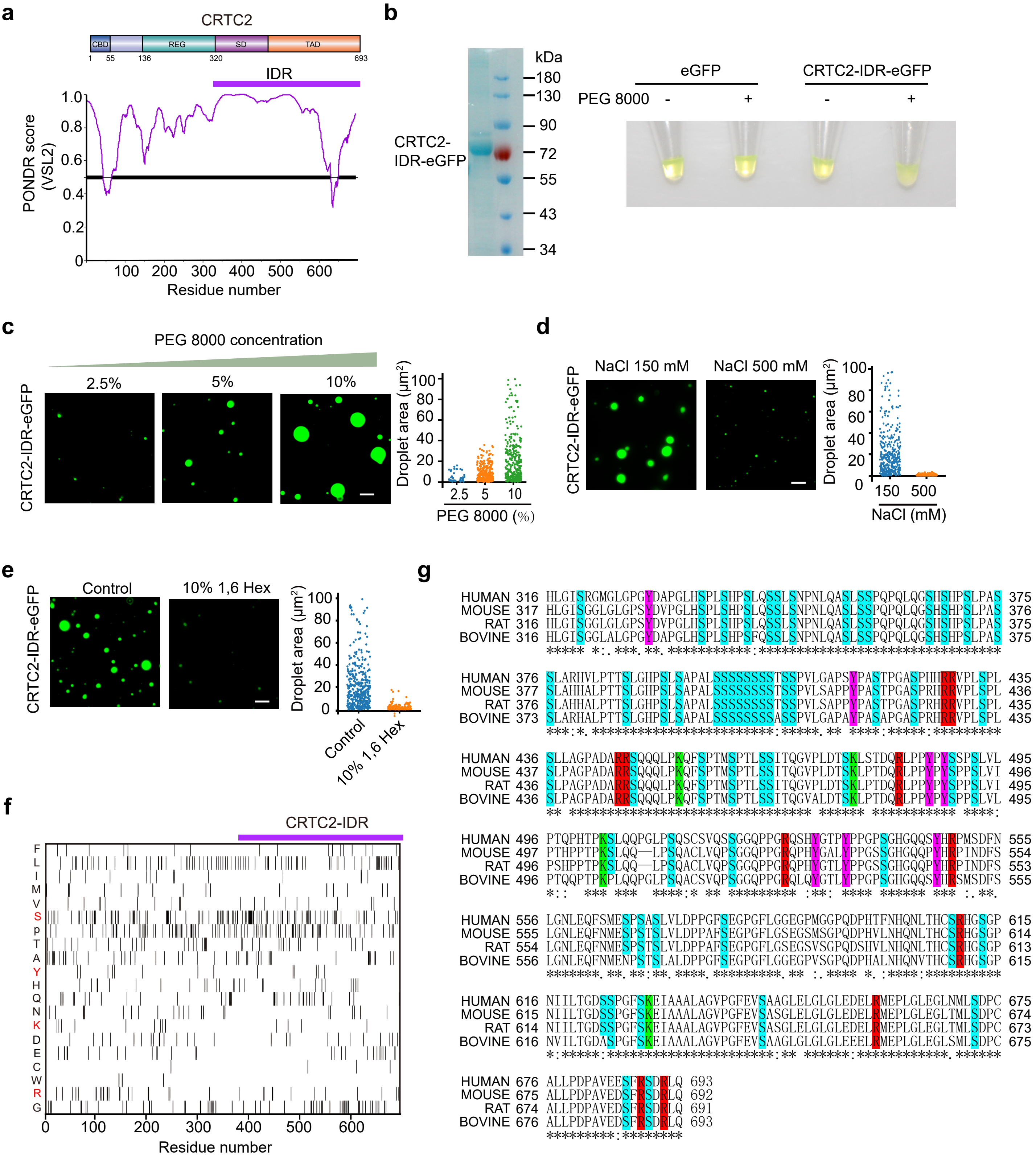

a
CRTC2
CBD
REG
SD
TAD
1
55
136
320
693
IDR
PONDR score (VSL2)
1.0
0.8
0.6
0.4
0.2
0
100
200
300
400
500
600
Residue number
b
kDa
180
130
90
72
55
43
34
CRTC2-IDR-eGFP
eGFP
CRTC2-IDR-eGFP
PEG 8000 - + - +
c
PEG 8000 concentration
2.5%
5%
10%
CRTC2-IDR-eGFP
Droplet area (μm²)
100
80
60
40
20
0
2.5
5
10
PEG 8000 (%)
d
NaCl 150 mM
NaCl 500 mM
CRTC2-IDR-eGFP
Droplet area (μm²)
100
80
60
40
20
0
150
500
NaCl (mM)
e
Control
10% 1,6 Hex
CRTC2-IDR-eGFP
Droplet area (μm²)
100
80
60
40
20
0
Control
10% 1,6 Hex
f
CRTC2-IDR
F
L
I
M
V
S
P
T
A
Y
H
Q
N
K
D
E
C
W
R
G
0
100
200
300
400
500
600
Residue number
g
HUMAN 316 HLGISRGMGLGPGYDAPGLHSPLSHPSLQSSLSNPNLQASLSSPQPQLQGSHSHPSLPAS 375
MOUSE 317 HLGISGGLGLGPSYDVPGLHSPLSHPSLQSSLSNPNLQASLSSPQPQLQGSHSHPSLPAS 376
RAT 316 HLGISGGLGLGPSYDVPGLHSPLSHPSLQSSLSNPNLQASLSSPQPQLQGSHSHPSLPAS 375
BOVINE 316 HLGISGGLALGPGYDAPGLHSPLSHPSFQSSLSNPNLQASLSSPQPQLQGSHSHPSLPAS 375
HUMAN 376 SLARHVLPTTSLGHPSLSAPALSSSSSSSSTSSPVLGAPSYPASTPGASPHHRRVPLSPL 435
MOUSE 377 SLAHHALPTTSLGHPSLSAPALSSSSSSSSTSSPVLSAPPYPASTPGASPRHRRVPLSPL 436
RAT 376 SLAHHALPTTSLGHPSLSAPALSSSSSSSSTSSPVLSAPPYPASTPGASPRHRRVPLSPL 435
BOVINE 373 SLARHALPTTSLGHPSLSAPALSSSSSSSSASSPVLGAPAYPASAPGASPRHRRVPLSPL 435
HUMAN 436 SLLAGPADARRSQQQLPKQFSPTMSPTLSSITQGVPLDTSKLSTDQRLPPYPYSSPSLVL 495
MOUSE 437 SLPAGPADARRSQQQLPKQFSPTMSPTLSSITQGVPLDTSKLPTDQRLPPYPYSPPSLVI 496
RAT 436 SLPAGPADARRSQQQLPKQFSPTMSPTLSSITQGVPLDTSKLPTDQRLPPYPYSPPSLVI 495
BOVINE 436 SLPAGPADARRSQQQLPKQFSPTMSPTLSSITQGVALDTSKLPTDQRLPPYPYSPPSLVL 495
HUMAN 496 PTQPHTPKSLQQPGLPSQSCSVQSSGGQPPGRQSHYGTPYPPGPSGHGQQSYHRPMSDFN 555
MOUSE 497 PTHPPTPKSLQQ—LPSQACLVQPSGGQPPGRQPHYGALYPPGSSGHGQQPYHRPINDFS 554
RAT 496 PSHPPTPKSLQQ—LPSQACLVQPSGGQPPGRQPHYGTLYPPGSSGHGQQPYHRPINDFS 553
BOVINE 496 PTQQPTPKPLQQPGLPSQACSVQPSGGQPPGRQLQYGTLYPPGPSGHGQQSYHRSMSDFS 555
HUMAN 556 LGNLEQFSMESPSASLVLDPPGFSEGPGFLGGEGPMGGPQDPHTFNHQNLTHCSRHGSGP 615
MOUSE 555 LGNLEQFNMESPSTSLVLDPPAFSEGPGFLGSEGSMSGPQDPHVLNHQNLTHCSRHGSGP 614
RAT 554 LGNLEQFNMESPSTSLVLDPPAFSEGPGFLGSEGSVSGPQDSHVLNHQNLTHCSRHGSGP 613
BOVINE 556 LGNLEQFNMENPSTSLALDPPGFSEGPGFLGGEGPVSGPQDPHALNHQNVTHCSRHGSGP 615
HUMAN 616 NIILTGDSSPGFSKEIAAALAGVPGFEVSAAGLELGLGLEDELRMEPLGLEGLNMLSDPC 675
MOUSE 615 NIILTGDSSPGFSKEIAAALAGVPGFEVSASGLELGLGLEDELRMEPLGLEGLTMLSDPC 674
RAT 614 NIILTGDSSPGFSKEIAAALAGVPGFEVSASGLELGLGLEDELRMEPLGLEGLTMLSDPC 673
BOVINE 616 NVILTGDASPGFSKEIAAALAGVPGFEVSAAGLGLGLGLEEELRMEPLGLEGLSMLSDPC 675
HUMAN 676 ALLPDPAVEESFRSDRLQ 693
MOUSE 675 ALLPDPAVEDSFRSDRLQ 692
RAT 674 ALLPDPAVEDSFRSDRLQ 691
BOVINE 676 ALLPDPAVEDSFRSDRLQ 693

**Figure S1 Characterization of CRTC2 condensation in vitro.** a) Graph revealing an intrinsic disorder region (IDR) for CRTC2. PONDR (predictor of natural disordered regions) VSL2 scores are shown on the y axis, and amino acid positions are shown on the x axis. The purple bar designates the IDR under investigation. b) Expression of CRTC2-IDR fused to eGFP (left). Visualization of turbidity associated with the indicated group (right). c) Representative images of droplet formation at different PEG 8000 concentrations (left). Quantification of the size of droplets (right). d) Representative images of droplet formation at different salt concentrations (left). Quantification of the size of droplets (right). e) Representative images of droplet formation with and without treatment with 10% 1,6-hexanediol (left). Quantification of the size of droplets (right). f) Amino acid composition of CRTC2. Each row represents information for a single amino acid, depicted (left) using the single-letter code. S, Y, K, R have been reported to be critical for phase separation. Black bars represent the occurrence of the indicated amino acid at that position in CRTC2. The purple bar represents the IDR of CRTC2 under investigation. g) Multiple species sequence alignment of the CRTC2 IDR. Conserved K, R, S, and Y residues in IDR are colored. Scale bar, 10 µm (c, d, e). All results are from more than three independent experiments.

**Figure S2**

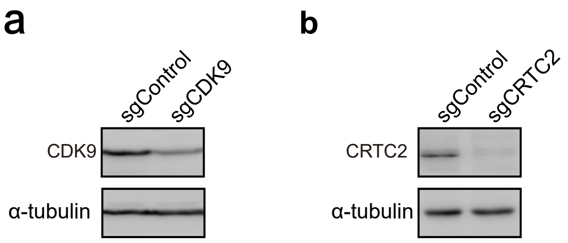


**Figure S2.** Generation of CDK9 KO and CRTC2 KO cell lines from 293T cells. Western blot analysis of whole-cell lysates from 293T cells infected with lentivirus carrying sgRNA against CDK9 (sgCDK9) (a) or CRTC2 (sgCRTC2) (b). All results are from more than three independent experiments.

## Figure S3

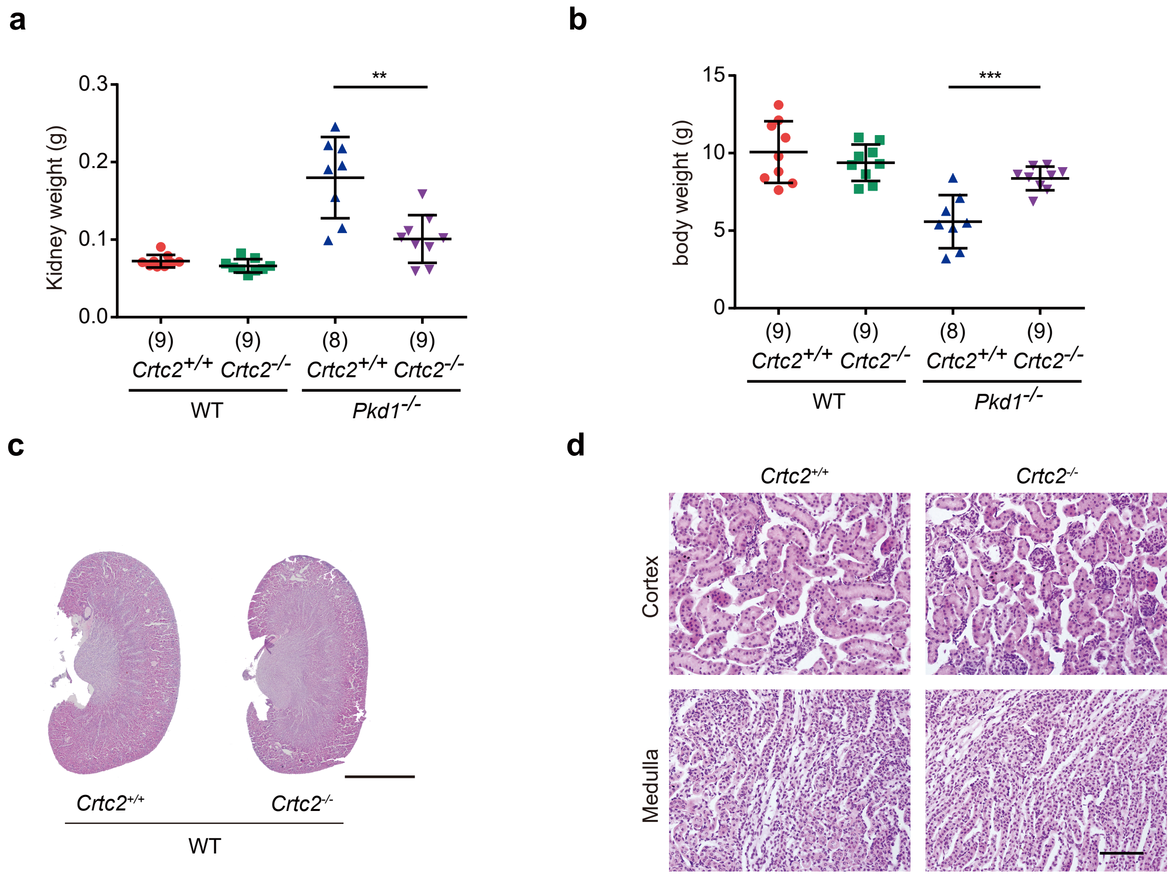


**Figure S3.** Supplementary data for animal experiments. a) The kidney weight of the indicated groups of mice . The number below each bar refers to the number of samples analyzed. b) The body weight of the indicated groups of mice . The number below each bar refers to the number of samples analyzed. c) Hematoxylin and eosin (H&E) staining of kidney sections from mice at P29 with indicated genotype. d) The structure of cortex and medulla from mice at 29 with indicated genotype. Data are presented as means ± SEM. The unpaired two-sided *t*-test was used for statistical analysis. $**P < 0.01$, $***P < 0.01$. Scale bar, 2 mm (a), 20 μm (d)

## Figure S4

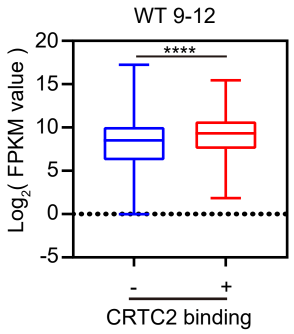


**Figure S4.** Correlation of CRTC2 binding with gene expression in human ADPKD cells. Boxplots of the normalized FPKM values of CRTC2-binding + and - gene sets in WT 9-12 cells. Data are presented as means ± SEM. The unpaired two-sided *t*-test was used for statistical analysis. ****$P < 0.0001$.

# Table S1

| Primer | Forward | Reverse |
|---|---|---|
| **7SK snRNA** | GACATCTGTCACCCCATTGA | GCGCAGCTACTCGTATACCC |
| **Creb5** | TAGCCTGCCCTAGTTTGGGT | AGAAAATCCAAAGCCGCTCG |
| **Smox** | TCTGCACAGAGATGCTTCGACAGT | TGAGCCCACCTGTGTGTAGGAAT |
| **Nnmt** | AGGAACCAGGAGCCTTTGACT | CCTGAGGGCAGTGCGATAGG |
| **Spock1** | TGCACGGACAAGGAGCTGCG | GAACCAGTCCTTCAGCCGG |
| **NNMT** | TGGCTTCTGGAGGAAAGAGA | AATCAGCAGGTCTCCCTTCA |
| **LDHA** | AGCCCGATTCCGTTACCT | CACCAGCAACATTCATTCCA |
| **PYGB** | ACGCAGCAGCACTACTAC | TCGCAGGCATTCTGAAGG |
| **PRNP** | AGAGGCCCAGGTCACTCC | GAGCTTCTCCTCTCCTCACG |

# Table S2

| Primer | Forward | Reverse |
|---|---|---|
| **LDHA-A** | GATGAGATGCCAGTGGGGTG | CCAAAACTCGACGCTGCTTT |
| **LDHA-B** | AACCTCCCCAGGTTTCATGG | GTGGAACAGCTATGCTGACG |
| **LDHA-C** | GTGCATTCCCGGTACGGTAG | GAACACATGCGTTGTGTGGG |
| **LDHA-D** | TCCAGTTCTCGGAGCCCATA | GCCCGAGATACACCAGTAGC |
| **LDHA-E** | AGGCCTCGTAACAACACAGG | AGGCCAGGAGCACCAATAAC |
| **LDHA-F** | TGTGAACGTTGAGCTTGGGG | CAGCCGTGATAATGACCAGC |
| **LDHA-G** | TTCAATTGGCTAGAGAAACAGGT | CTGGGTGCAGAGTCTTCAGAG |
| **LDHA-H** | GGCTACACATCCTGGGCTAT | GCATTCAAATGCAGCGTATCAC |
| **PLAU-A** | CAGCGGGTGCAACTTAAGAG | AGACAGAACGCTGTGAAGGG |
| **PLAU-B** | AGCGCATGGATAAGGAAGTTCT | CCGTTATGAAGCGTGACACC |
| **PLAU-C** | AGCAAGGTTGGCTCTGAAGC | CCCAGTAGGACTCGCTCTCT |
| **PLAU-D** | TATCTGGGGACTGCCACTGA | GCCCCAACTTGCCTAAGACT |
| **PLAU-E** | CCAGCGAACTGTGACTGTCT | TAACTCCTGCCTGCTCTTGC |
| **PLAU-F** | CTCTTCGACTCTTCTGCCCC | ACTTCATCTCCCCTTGCGTG |
| **PLAU-G** | AGCTACTTCCTCGGCACTTG | TTGATCACGCTGGGCTCATT |
| **PLAU-H** | CCAGGTGAGTGTTCCAAGCA | TGAAGTGACTAGCGCAGAGC |
